# Supplementary material for: Lifetime Weight Course as a Phenotypic Marker of Severity and Therapeutic Response in Patients with Eating Disorders
Source: Nutrients. 2021 Jun 13;13(6):2034. doi: 10.3390/nu13062034 (PMC8231878; doi:10.3390/nu13062034)
Supplement: Supplementary file 1 [file nutrients-13-02034-s001.zip › nutrients-1222319-supplementary.pdf]

Figure S1. (supplementary material). Flow-chart with the sampling procedure. Note. AN: anorexia nervosa. BN: bulimia nervosa. BED: binge eating disorder. OSFED: other specified feeding or eating disorder.

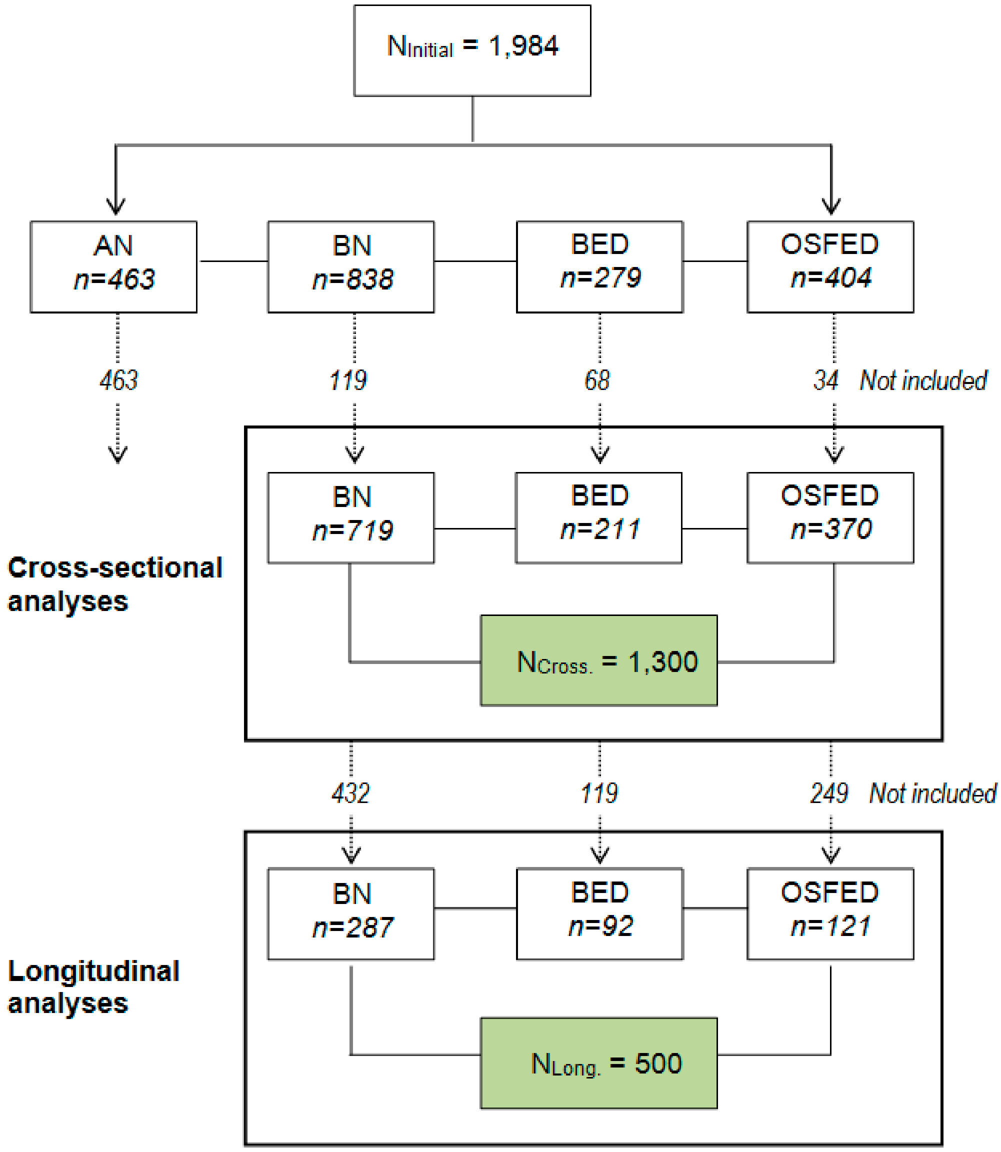

Table S1. Comparison between the groups included and not included in the substudy 2 of treatment outcomes.

		Excluded n=800		Participants n=500			
		**n**	**%**	**n**	**%**	**p**	**|h|**
BMI group	Obese-obese	45	5.6%	29	5.8%	.715	0.01
	Obese-normo	96	12.0%	60	12.0%		0.00
	Normo-normo	474	59.3%	282	56.4%		0.06
	Nomo-obese	185	23.1%	129	25.8%		0.06
Diagnosis	BN	432	54.0%	287	57.4%	**.017***	0.07
	BED	119	14.9%	92	18.4%		0.09
	OSFED	249	31.1%	121	24.2%		0.16
Sex	Female	760	95.0%	458	91.6%	**.014***	0.14
	Male	40	5.0%	42	8.4%		0.14
Education level	Primary	379	47.4%	202	40.4%	**.023***	0.14
	Secondary	318	39.8%	213	42.6%		0.06
	University	103	12.9%	85	17.0%		0.12
Civil status	Single	578	72.3%	354	70.8%	.519	0.03
	Married/Partner	160	20.0%	112	22.4%		0.06
	Divorced/separated	62	7.8%	34	6.8%		0.04
Employment	Unemployed	298	37.3%	152	30.4%	**.014***	0.15
	Student	288	36.0%	182	36.4%		0.01
	Employed	214	26.8%	166	33.2%		0.14
		Mean	SD	Mean	SD	p	|d|
Age (years-old)		29.23	10.65	28.44	9.68	.177	0.08
Onset of ED		19.75	8.00	20.57	8.20	.075	0.10
EDI-2 total		104.69	40.19	105.73	41.41	.653	0.03
SCL-90R GSI		1.79	0.73	1.77	0.71	.600	0.03

Note. BN: bulimia nervosa. BED: binge eating disorder. OSFED: other specified feeding and eating disorder. SD: standard deviation. *Bold: significant comparison. †Bold: effect size into the mild/moderate (|d|>0.50 or |h|>0.50) to large/good range (|d|>0.80 or |h|>0.80).
